# Fluvastatin use and risk of acute pancreatitis: a population-based case-control study in Taiwan

**DOI:** 10.1051/bmdcn/2017070317

**Published:** 2017-08-25

**Authors:** Kuan-Fu Liao, Po-Tsung Huang, Ching-Chun Lin, Cheng-Li Lin, Shih-Wei Lai

**Affiliations:** 1 College of Medicine, Tzu Chi University Hualien 970 Taiwan; 2 Department of Internal Medicine, Taichung Tzu Chi General Hospital Taichung 427 Taiwan; 3 Graduate Institute of Integrated Medicine, China Medical University Taichung 404 Taiwan; 4 College of Medicine, China Medical University Taichung 404 Taiwan; 5 Department of Family Medicine, China Medical University Hospital Taichung 404 Taiwan; 6 Management Office for Health Data, China Medical University Hospital Taichung 404 Taiwan

**Keywords:** Acute pancreatitis, Alcohol, Biliary stone, Diabetes mellitus, Fluvastatin

## Abstract

Objectives: This study aimed to examine the association between fluvastatin use and acute pancreatitis in Taiwan.

Methods: Using the database from the Taiwan National Health Insurance (NHI) Program, we designed a case-control study which consisted of 3501 individuals aged 20-84 with new at-the-time diagnoses acute pancreatitis as the case group and 8373 randomly selected individuals without acute pancreatitis as the control group during the period of 1998-2011. Both groups were matched for sex, age, and index year of being diagnosed with acute pancreatitis. “Current use” of fluvastatin was defined as individuals whose last remaining tablet of fluvastatin was noted ≤ 7 days before the date of their being diagnosed with acute pancreatitis. “Late use” of fluvastatin was defined as individuals whose last remaining tablet of fluvastatin was noted within 8-30 days before the date of their being diagnosed with acute pancreatitis. “No use” of fluvastatin was defined as individuals who had never had a fluvastatin prescription. The odds ratio (OR) and 95% confidence interval (CI) for acute pancreatitis associated with fluvastatin use was examined using a multivariable unconditional logistic regression analysis.

Results: After adjustment for potential confounders, the multivariable analysis showed that the adjusted ORs of acute pancreatitis were 1.17 for individuals with “current use” of fluvastatin (95% CI 0.69, 1.97) and 1.82 for individuals with “late use” of fluvastatin (95% CI 0.41, 8.19), but there was no statistical significance when compared with individuals with “no use” of fluvastatin.

Conclusions: In this this study, no association was detected between fluvastatin use and acute pancreatitis.

## 1. Introduction

Fluvastatin, being a member of the statins, is the first fully synthetic HMG-CoA reductase inhibitor and is commonly used to treat patients with hypercholesterolemia and further to reduce the risk of cardiovascular disease [1, 2]. Clinically, fluvastatin has been used with relative efficacy, safety, and tolerability. Its few adverse effects include myopathy (including myositis and rhabdomyolysis), as well as asymptomatic, reversible elevations of serum creatine phosphokinase and serum hepatic transaminase [1, 3, 4]. However, one case regarding fluvastatin-related acute pancreatitis has been reported by Tysk *et al.* in Saudi-Arabia [5]. In addition, the U.S. Food and Drug Administration (FDA) has also reported that during 2010, only 6 individuals (0.97%) had acute pancreatitis among the 617 individuals who reported having adverse effects when using fluvastatin [6].

Drug-induced acute pancreatitis contributes to approximately 2% of the cases of acute pancreatitis [7, 8]. To date, no population-based pharmacoepidemiological research is available on the association between fluvastatin use and acute pancreatitis. Given extensive use of fluvastatin clinically and on the basis of the above-mentioned case report and the U.S. FDA report, we made a plausible hypothesis that there is an association between fluvastatin use and acute pancreatitis. If this association is shown to really exist, physicians can then alert their patients as to the risk of acute pancreatitis when using fluvastatin. It is with this in mind that we conducted a population-based case-control study to examine this question.

## 2. Methods

### 2.1. Study design and study population

Taiwan is an independent country with more than 23 million people. Using the database of the Taiwan National Health Insurance (NHI) Program, we designed a population-based case-control study. Briefly, this insurance program started in March 1, 1995, and now covers approximately 99% of the 23 million people living in Taiwan [9]. Details of the NHI program have been well-reported previously [10–21]. This study was approved by the Ethics Review Board of China Medical University and Hospital in Taichung, Taiwan (CMUH-104-REC2-115).

### 2.2. Inclusion criteria

Based on the International Classification of Diseases 9th Revision-Clinical Modification (ICD-9 code), we defined the cases of acute pancreatitis for our study as individuals aged 20-84 years at the date of their diagnosis, who were then newly diagnosed with acute pancreatitis (ICD-9 code 577.0) during the period of 1998-2011. We defined the index date for each case as the date of their being diagnosed acute pancreatitis. We randomly selected individuals without acute pancreatitis from the same database as controls, and matched them to those with acute pancreatitis according sex, age (within 5 years), and the index year of the latter’s being diagnosed with acute pancreatitis. Individuals with chronic pancreatitis (ICD-9 code 577.1) or pancreatic cancer (ICD-9 code 157) before the date of their being diagnosed with acute pancreatitis were excluded from this study. Individuals who received at least 1 prescription for other statins or non-statin lipid-lowering drugs were also excluded from the study.

### 2.3. Comorbidities potentially associated with acute pancreatitis

Comorbidities before the date of an individual being diagnosed with acute pancreatitis were included as follows: alcohol-related diseases (ICD-9 codes 291, 303, 305.00, 305.01, 305.02, 305.03, 571.0-571.3, 790.3 and V11.3), biliary stone (ICD-9 code 574), cardiovascular diseases including coronary artery disease, heart failure, cerebrovascular disease, and peripheral atherosclerosis (ICD-9 codes 410-414, 428, 430-438 and 440-448), chronic kidney disease (ICD-9 codes 585-586 and 588.8-588.9), chronic obstructive pulmonary disease (ICD-9 codes 491, 492, 493 and 496), diabetes mellitus (ICD-9 code 250), hepatitis B (ICD-9 codes V02.61, 070.20, 070.22, 070.30 and 070.32), hepatitis C (ICD-9 codes V02.62, 070.41, 070.44, 070.51 and 070.54), hyperparathyroidism (ICD-9 code 252.0), and hypertriglyceridemia (ICD-9 codes 272.1, 272.2 and 272.4). The diagnostic accuracy of these comorbidities based on ICD-9 codes has been written about in previous studies [22–30].

### 2.4. Definition of fluvastatin exposure

According to its prescription date, the last remaining tablet of fluvastatin can be estimated. To decrease biased results, individuals whose last remaining tablet of fluvastatin was noted > 1 month before the date of their being diagnosed with acute pancreatitis were excluded from the study. Therefore, only individuals whose last remaining tablet of fluvastatin was noted within 1 month prior to the date of their being diagnosed with acute pancreatitis were included in this study. The elimination half-life of fluvastatin ranges from approximately 30 minutes to 1.2 hours in healthy individuals [3, 31]. Thus, a period of 7 days was used as a cutoff point, something which we adopted from previous studies [27, 28, 32]. “Current use” of fluvastatin was defined as individuals whose last remaining tablet of fluvastatin was noted ≤ 7 days before the date of their being diagnosed with acute pancreatitis or those still having fluvastatin tablets at the date of their being diagnosed with acute pancreatitis. “Late use” of fluvastatin was defined as individuals whose last remaining tablet of fluvastatin was noted within 8-30 days before the date of their being diagnosed with acute pancreatitis. “No use” of fluvastatin was defined as individuals who never had a fluvastatin prescription.

### 2.5. Statistical Analysis

The differences in sex, age, fluvastatin use, and comorbidities between the cases and the controls were compared using a *Chi- square* test and a Fisher exact test for categorized variables, and a f-test for continuous variables. Initially, all variables were included in a univariable unconditional logistic regression model. Variables found to be significant in this univariable model were then included in a multivariable unconditional logistic regression model to measure the odds ratio (OR) and 95% confidence interval (CI) for acute pancreatitis risk associated with fluvastatin use and comorbidities. We used the SAS software for all data analysis (version 9.2 for Windows; SAS Institute Inc., Cary, North Carolina, USA). A *P* value < 0.05 was considered statistically significant.

## 3. Results

### 3.1. Demographic characteristics of the study population

In total, 3501 individuals with acute pancreatitis were selected as cases and 8373 individuals without acute pancreatitis were selected as controls. Table 1 presents the demographic characteristics between the cases and controls. The mean ages (standard deviation) were 49.2 (16.0) years in cases and 48.6 (15.8) years in controls (f-test, *P* = 0.03). The cases were more likely to have higher proportions of “current use” of fluvastatin and “late use” of fluvastatin than the controls, but that was without a statistical significance (0.66% vs. 0.49% for current use, and 0.09% vs. 0.05% for late use, respectively, Chi-square test, *P* = 0.39). The individuals in the cases group tended to have higher proportions of alcohol-related diseases, biliary stone, cardiovascular disease, chronic kidney disease, chronic obstructive pulmonary disease, diabetes mellitus, hepatitis B, hepatitis C, hyperparathyroidism, and hypertriglyceridemia than the individuals in the controls (*Chi-* square test, *P* < 0.05 for all).

**Table 1:** Demographic characteristics of cases with acute pancreatitis and controls in Taiwan during the period of 1998-2011.

	Controls N = 8373		Cases N = 3501		
Variable	n	(%)	n	(%)	*P* value*
Sex					0.99
Female	2679	(32.0)	1120	(32.0)	
Male	5694	(68.0)	2381	(68.0)	
Age group (years)					0.07
20-39	2979	(35.6)	1203	(34.4)	
40-64	3820	(45.6)	1578	(45.1)	
65-84	1574	(18.8)	720	(20.6)	
Age (years), mean (standard deviation)†	48.6	(15.8)	49.2	(16.0)	0.03
Fluvastatin					0.39
No use	8328	(99.46)	3475	(99.25)	
Current use	41	(0.49)	23	(0.66)	
Late use	4	(0.05)	3	(0.09)	
Comorbidities before index date					
Alcohol-related diseases	1148	(13.7)	545	(15.6)	0.008
Biliary stone	715	(8.54)	406	(116)	< 0.001
Cardiovascular diseases	1417	(16.9)	666	(19.0)	0.006
Chronic kidney disease	115	(1.37)	100	(2.86)	< 0.001
Chronic obstructive pulmonary disease	1123	(13.4)	519	(14.8)	0.04
Diabetes mellitus	623	(7.44)	365	(10.4)	< 0.001
Hepatitis B	185	(2.21)	146	(4.17)	< 0.001
Hepatitis C	79	(0.94)	93	(2.66)	< 0.001
Hyperparathyroidism&	1	(0.01)	9	(0.26)	<0.001
Hypertriglyceridemia	658	(7.86)	336	(9.60)	0.002

Data are presented as the number of subjects in each group with percentages given in parentheses, or mean with standard deviation given in parentheses.

* Chi-square test,

& Fisher exact test, and

† *t* test comparing subjects with and without acute pancreatitis.

### 3.2. Association between acute pancreatitis and fluvastatin use

After adjusting for multiple confounders, the multivariable analysis showed that the adjusted ORs of acute pancreatitis were 1.17 for individuals with “current use” of fluvastatin (95%CI 0.69, 1.97) and 1.82 for individuals with “late use” of fluvastatin (95% CI 0.41, 8.19), but there was no statistical significance when compared with individuals with “no use” of fluvastatin (Table 2).

**Table 2:** Crude and adjusted odds ratio and 95% confidence interval for acute pancreatitis associated with fluvastatin use in Taiwan during the period of 1998-2011.

	Crude		Adjusted†	
Variable	OR	(95% CI)	OR	(95% CI)
Fluvastatin (no use as a reference)				
Current use	1.34	(0.81, 2.24)	1.17	(0.69, 1.97)
Late use	1.80	(0.40, 8.04)	1.82	(0.41, 8.19)

† Variables found to be significant in the univariable unconditional logistic regression model were further analyzed in the multivariable unconditional logistic regression model.

Additionally adjusted for age, alcohol-related diseases, biliary stone, cardiovascular diseases, chronic kidney disease, chronic obstructive pulmonary disease, diabetes mellitus, hepatitis B, hepatitis C, hyperparathyroidism, and hypertriglyceridemia.

## 4. Discussion

In this population-based case-control study, we observed that fluvastatin use was not associated with acute pancreatitis, no matter whether “current use” or “late use” of fluvastatin. To date, only one case regarding fluvastatin-related acute pancreatitis has been reported by Tysk *et al.* in Saudi-Arabia, a case which was definitely confirmed after receiving a re-challenge test [5]. The adverse effects of fluvastatin use published by the U.S. Food and Drug Administration (FDA) do not indicate a causal relationship between fluvastatin use and acute pancreatitis [6]. However, besides this study no other pharmacoepidemiological research on this issue is available. We cannot compare our research with anything. Therefore, this present study cannot support the hypothesis of fluvastatin-related acute pancreatitis raised in the one aforementioned case report and the adverse effect report issued by the U.S. FDA.

The relevant literature was reviewed to address the biological plausibility of fluvastatin-related or -caused acute pancreatitis. Generally speaking, serious adverse effects of statin therapy are commonly associated with concomitant drugs affecting statin metabolism and are rarely associated with specific adverse responses to statin monotherapy [2, 33–35]. Fluvastatin is mainly metabolized by the cytochrome P-450 2C9 pathway, not by the cytochrome P-450 3A4 pathway [2, 33–35]. In theory, fluvastatin is less likely to provoke drug interaction than other statins [2, 33–35]. Whether some concomitant key drugs potentially inhibiting cytochrome P-450 2C9 might provoke drug interaction with fluvastatin should be considered. Despite serious adverse effects of statin therapy, such effects are rarely associated with statin monotherapy. Whether fluvastatin has an idiosyncratic effect or a direct toxic effect on the pancreas should also be considered. More research on this issue is needed to elucidate this issue.

Some concerns of this present study should be taken into consideration when interpreting our results. First, since the Taiwan NHI claims database is inherently restricted by a lack of laboratory results, diagnostic misclassification of acute pancreatitis is likely, which may have confounded our results. However, the diagnostic accuracy of acute pancreatitis based on ICD-9 codes has been examined in previous studies [36-39]. Second, due to the same limitation, some crucial confounders such as detrimental life style or unhealthy habits are not documented in this database. Because of this, we used alcohol-related diseases instead of alcoholism and chronic obstructive pulmonary disease instead of cigarette smoking. Third, and also due to the same limitation, we are not sure how many individuals with acute pancreatitis had said acute pancreatitis caused by fluvastatin use in this study because the etiologies of acute pancreatitis are not documented in this database. In addition, only 0.75% of the cases in the database had been exposed to fluvastatin. Insufficient statistical power is a great concern. Fourth, only one case regarding fluvastatin-related acute pancreatitis has been reported in the extant literature [5], and only 6 cases of fluvastatin-related acute pancreatitis have been reported by the U.S. Food and Drug Administration [6]. The incidence of fluvastatin-related or -caused acute pancreatitis might be very low. Furthermore, no statistically significant findmgs have been disclosed. Whether the frequency of this adverse effect is really so low or only under-reported cannot be determined in this study. Fifth, we hope to show the original difference of comorbidities between the cases and controls. Only sex and age were matched between the cases and controls; the comorbidities were not matched. That is why the case group tended to have higher proportions of alcohol-related diseases, biliary stone, cardiovascular diseases, chronic kidney disease, chronic obstructive pulmonary disease, diabetes mellitus, hepatitis B, hepatitis C, hyperparathyroidism, and hypertriglyceridemia than the control group *(Chi*-square test, *P* < 0.05 for all). Last, although patients with acute pancreatitis may have recurrent attacks, only those with newly (at-the-time) diagnosed acute pancreatitis could be included in this study. Individuals with at-the-time current acute pancreatitis or chronic pancreatitis were not included.

Having said all of that, there are some advantages of this study. Individuals receiving at least 1 prescription for other statins or non-statin lipid-lowering drugs were excluded from this study. Therefore, this is the first population-based case-control study focusing on the association between only fluvastatin use and acute pancreatitis—it is not confounded by other lipidlowering drugs. In addition, this topic is clinically relevant with a potential implication for practice. The methodology and the analysis were well-conducted. The results are of clinical importance. The study provides updated pharmacoepidemiological evidence on this issue.

All things considered, we have concluded that no association can be detected between fluvastatin use and acute pancreatitis, regardless of whether the case is “current use” or “late use” of fluvastatin. Of course, additional studies are needed to confirm our findings.

## References

[1] Langtry HD, Markham A. Fluvastatin: a review of its use in lipid disorders. Drugs. 1999; 57: 583–606. 10235694 10.2165/00003495-199957040-00009

[2] Scripture CD, Pieper JA. Clinical pharmacokinetics of fluvastatin. Clin Pharmacokinet. 2001; 40: 263–281. 11368292 10.2165/00003088-200140040-00003

[3] Garnett WR. A review of current clinical findings with fluvastatin. Am J Cardiol. 1996; 78: 20–25. 10.1016/s0002-9149(96)00658-3 8875971

[4] Weiss RJ. Fluvastatin titrate-to-goal clinical practice study. Am J Ther. 1998; 5: 281–285. 10099070 10.1097/00045391-199809000-00002

[5] Tysk C, Al-Eryani AY, Shawabkeh AA. Acute pancreatitis induced by fluvastatin therapy. J Clin Gastroenterol. 2002; 35: 406–408. 12394230 10.1097/00004836-200211000-00010

[6] eHealthMe study from FDA and social media reports. Review: could fluvastatin cause acute pancreatitis? http://www.ehealthme.com/print/ds14688387. [cited in 2016 April 10]

[7] Wilmink T, Frick TW. Drug-induced pancreatitis. Drug Saf. 1996; 14: 406–423. 8828018 10.2165/00002018-199614060-00006

[8] Trivedi CD, Pitchumoni CS. Drug-induced pancreatitis: an update. J Clin Gastroenterol. 2005; 39: 709–716. 16082282 10.1097/01.mcg.0000173929.60115.b4

[9] National Health Insurance Research Database, Taiwan. http://nhird.nhri.org.tw/en/index.html. [cited in 2016 April 1, English version].

[10] Lai SW, Liao KF, Liao CC, Muo CH, Liu CS, Sung FC. Polypharmacy correlates with increased risk for hip fracture in the elderly: a population-based study. Medicine (Baltimore). 2010; 89: 295–299. 20827106 10.1097/MD.0b013e3181f15efc

[11] Hung SC, Liao KF, Lai SW, Li CI, Chen WC. Risk factors associated with symptomatic cholelithiasis in Taiwan: a population-based study. BMC Gastroenterol. 2011; 11: 11–111. 21999925 10.1186/1471-230X-11-111 PMC3215644

[12] Chen HY, Lai SW, Muo CH, Chen PC, Wang IJ. Ethambutol- induced optic neuropathy: a nationwide population-based study from Taiwan. Br J Ophthalmol. 2012; 96: 1368–1371. 22960095 10.1136/bjophthalmol-2012-301870

[13] Cheng KC, Chen YL, Lai SW, Mou CH, Tsai PY, Sung FC. Patients with chronic kidney disease are at an elevated risk of dementia: a population-based cohort study in Taiwan. BMC Nephrol. 2012; 13: 1471–2369. 10.1186/1471-2369-13-129 PMC3507799 23020192

[14] Cheng KC, Chen YL, Lai SW, Tsai PY, Sung FC. Risk of esophagus cancer in diabetes mellitus: a population-based case-control study in Taiwan. BMC Gastroenterol. 2012; 12: 12–177. 23234272 10.1186/1471-230X-12-177 PMC3531311

[15] Lai HC, Chang SN, Lin CC, Chen CC, Chou JW, Peng CY, *et al* Does diabetes mellitus with or without gallstones increase the risk of gallbladder cancer? Results from a population-based cohort study. J Gastroenterol. 2013; 48: 856–865. 23065035 10.1007/s00535-012-0683-z

[16] Lai HC, Tsai IJ, Chen PC, Muo CH, Chou JW, Peng CY, *et al* Gallstones, a cholecystectomy, chronic pancreatitis, and the risk of subsequent pancreatic cancer in diabetic patients: a population-based cohort study. J Gastroenterol. 2013; 48: 721–727. 23053420 10.1007/s00535-012-0674-0

[17] Chen YL, Cheng KC, Lai SW, Tsai IJ, Lin CC, Sung FC, *et al* Diabetes and risk of subsequent gastric cancer: a population-based cohort study in Taiwan. Gastric Cancer. 2013; 16: 389–396. 23053824 10.1007/s10120-012-0197-7

[18] Lai HC, Lin CC, Cheng KS, Kao JT, Chou JW, Peng CY, *et al* Increased incidence of gastrointestinal cancers among patients with pyogenic liver abscess: a population-based cohort study. Gastroenterology. 2014; 146: 129–137. 24095786 10.1053/j.gastro.2013.09.058

[19] Hung SC, Lai SW, Tsai PY, Chen PC, Wu HC, Lin WH, *et al* Synergistic interaction of benign prostatic hyperplasia and prostatitis on prostate cancer risk. Br J Cancer. 2013; 108: 1778–1783. 23612451 10.1038/bjc.2013.184 PMC3658521

[20] Yang SP, Muo CH, Wang IK, Chang YJ, Lai SW, Lee CW, *et al* Risk of type 2 diabetes mellitus in female breast cancer patients treated with morphine: A retrospective population-based time-dependent cohort study. Diabetes Res Clin Pract. 2015; 110: 285–290. 26515910 10.1016/j.diabres.2015.10.005

[21] Kuo SC, Lai SW, Hung HC, Muo CH, Hung SC, Liu LL, *et al* Association between comorbidities and dementia in diabetes mellitus patients: population-based retrospective cohort study. J Diabetes Complications. 2015; 29: 1071–1076. 26233574 10.1016/j.jdiacomp.2015.06.010

[22] Lai SW, Muo CH, Liao KF, Sung FC, Chen PC. Risk of acute pancreatitis in type 2 diabetes and risk reduction on anti-diabetic drugs: a population-based cohort study in Taiwan. Am J Gastroenterol. 2011; 106: 1697–1704. 21577242 10.1038/ajg.2011.155

[23] Liao KF, Lai SW, Li CI, Chen WC. Diabetes mellitus correlates with increased risk of pancreatic cancer: a population-based cohort study in Taiwan. J Gastroenterol Hepatol. 2012; 27: 709–713. 21929650 10.1111/j.1440-1746.2011.06938.x

[24] Hung SC, Hung SR, Lin CL, Lai SW, Hung HC. Use of celecoxib correlates with increased relative risk of acute pancreatitis: a case-control study in Taiwan. Am J Gastroenterol. 2015; 110: 1490–1496. 26323189 10.1038/ajg.2015.259

[25] Liao K-F, Lin C-L, Lai S-W. Schizophrenia Correlates with Increased Risk of Hepatocellular Carcinoma in Men: A Cohort Study in Taiwan. International Medical Journal. 2015; 22.

[26] Lai SW, Lai HC, Lin CL, Liao KF. Finasteride use and acute pancreatitis in Taiwan. J Clin Pharmacol. 2015; 55: 657–660. 25573785 10.1002/jcph.462

[27] Shen ML, Liao KF, Tsai SM, Lin CL, Lai SW. Herpes zoster correlates with pyogenic liver abscesses in Taiwan. Biomedicine-Taiwan. 2016; 6: 24–29. 10.7603/s40681-016-0022-4 PMC5112182 27854050

[28] Cheng KC, Lin WY, Liu CS, Lin CC, Lai HC, Lai SW. Association of different types of liver disease with demographic and clinical factors. Biomedicine-Taiwan. 2016; 6: 16–22. 10.7603/s40681-016-0016-2 PMC4996334 27518399

[29] Liao K-F, Lin C-L, Lai S-W, Chen W-C. Parkinson’s disease and risk of pancreatic cancer: a population-based case-control study in Taiwan. Neurology Asia. 2015; 20.

[30] Lin HF, Lai SW, Lin WY, Liu CS, Lin CC, Chang CM. Prevalence and factors of elevated alanine aminotransferase in central Taiwan - a retrospective study. Biomedicine-Taiwan. 2016; 6: 25–30. 10.7603/s40681-016-0011-7 PMC4864771 27161001

[31] Smith HT, Jokubaitis LA, Troendle AJ, Hwang DS, Robinson WT. Pharmacokinetics of fluvastatin and specific drug interactions. Am J Hypertens. 1993; 6: 375S–382S. 8297546 10.1093/ajh/6.11.375s

[32] Lai SW, Lin CL, Liao KF. Atorvastatin Use Associated With Acute Pancreatitis: A Case-Control Study in Taiwan. Medicine. 2016; 95: e2545. 26886597 10.1097/MD.0000000000002545 PMC4998597

[33] Sica DA, Gehr TW. Rhabdomyolysis and statin therapy: relevance to the elderly. Am J Geriatr Cardiol. 2002; 11: 48–55. 11773716 10.1111/j.1076-7460.2002.01422.x

[34] Corsini A. The use of statins in optimising reduction of cardiovascular risk: focus on fluvastatin. Int J Clin Pract. 2004; 58: 494–503. 15206507 10.1111/j.1368-5031.2004.00154.x

[35] Neuvonen PJ, Backman JT, Niemi M. Pharmacokinetic comparison of the potential over-the-counter statins simvastatin, lovastatin, fluvastatin and pravastatin. Clin Pharmacokinet. 2008; 47: 463–474. 18563955 10.2165/00003088-200847070-00003

[36] Lai SW, Lin CL, Liao KF. Use of methimazole and risk of acute pancreatitis: A case-control study in Taiwan. Indian J Pharmacol. 2016; 48: 192–195. 27127323 10.4103/0253-7613.178841 PMC4825438

[37] Hung SC, Liao KF, Hung HC, Lin CL, Lai SW, Lin CH. Nabume- tone use and risk of acute pancreatitis in a case-control study. Pancreatology. 2016; 16: 353–357. 27029853 10.1016/j.pan.2016.03.003

[38] Liao KF, Cheng KC, Lin CL, Lai SW. Etodolac and the risk of acute pancreatitis. Biomedicine-Taiwan. 2017; 7: 25–29. 10.1051/bmdcn/2017070104 PMC5439338 28474580

[39] Lai S-W, Lin H-F, Lin C-L, Liao K-F. No association between losartan use and acute pancreatitis in hypertensive patients. European Journal of Hospital Pharmacy. 2016; ejhpharm-2015-000840. 10.1136/ejhpharm-2015-000840 PMC6451466 31156917

